# Impact of Interprofessional Relationships from Nurses’ Perspective on the Decision-Making Capacity of Patients in a Clinical Setting

**DOI:** 10.3390/ijerph15010049

**Published:** 2017-12-29

**Authors:** Jesús Molina-Mula, Julia Gallo-Estrada, Catalina Perelló-Campaner

**Affiliations:** 1 Nursing and Physiotherapy Department, University of Balearic Islands, 07122 Palma de Mallorca, Spain; j.gallo@uib.es; 2 Emergency Care Service 061, 07014 Palma de Mallorca, Spain; c.perello@uib.es

**Keywords:** decision making, interprofessional relations, nurse-patient relations, Foucault

## Abstract

Interprofessional relationships may impact the decision making of patients in a clinical setting. The objective of this study was to analyse the decision-making capabilities of patients from nurses’ perspectives of interprofessional relationships using Foucauldian ethics. This qualitative study was based on poststructuralist Foucault references with in-depth interviews of nurses working in internal medicine and specialties in a general hospital. The patients constantly appeared in the definition of teamwork, but also as a passive element used by every professional to communicate with others. Nurses continue modelling a type of patient passivity, or what Foucault called passive subjectivity in relation to oneself, because the patient is guided and directed to take charge of a truth provided by professionals. Nurses must break the rigid design of sections or professional skills, and adopt a model of teamwork that meets the needs of the patient and increases their decision-making power. The quality of care will increase to the extent that professionals establish a relationship of equality with the patient, allowing the patient to make real decisions about their care. An egalitarian model of teamwork is beneficial to the patient, abandoning the idea of a team where the patient and family are constantly excluded from decisions about their care.

## 1. Introduction

The ethical proposal of Michel Foucault involves the idea of a plurality of individual models, or of a diversity of spaces and conditions, that enable the construction of a range of forms of subjectivity, in which the freedom to be thought of in another way exists [1]. Foucault’s work starts from an expression of scepticism about the idea of a Kantian subject, universal and founding. It asserts that certain truth games, discourses, and practices determine the construction of models of subjectivity that are fragile and unstable with constant transformation. Foucault [2] tries to demonstrate his rejection of a constituent subject with this fragility.

The ethical individual is not universal or suprahistorical, but is rather a reality influenced by structures, experiences with themselves, and is permeable to the changes that affect them by producing self-training [3]. Therefore, the ethical individual is an element susceptible to self-constitution and self-conduction in health institutions.

Additionally, in a healthcare environment, a close relationship exists between this ethical individual and the power. Through a series of control and normalization structures or devices of domination, used by professionals and the institution, the behaviours and attitudes that patients must assume are dictated. These exercises of dominant power are what Foucault called the “ethical question” [4,5,6].

Foucault’s ethical perspective helps to understand how the autonomy of the patient is articulated in decision making. Foucault abandons the Kantian idea of the subject as a phenomenon or existence, and configures the subject through the relationship of power and knowledge in a historical and social context. This process by which the subject is constructed, Foucault called “subjectivation” [7]. From this perspective, the patient, when entering a health institution, is reconfigured as a subject under the control of the professionals and the dynamics of the organization [3]. Thus, in a clinical context, subjectivation is normalized and is incorporated into the system’s objectives of productivity, performance, and functionality; and the patient is subjected to the imperative of inner or psychological truth as an expression of ultimate essence or foundation [1].

Foucault, through criticism of several elements, describes the genealogy of ethics: power relations, archaeology of knowledge and truth, discourses, the relationship between power, knowledge, and truth, and government. The genealogy of ethics corresponds to the description of the modern mechanisms of appropriation and control of individuals that block the space of ethical relationship. The disciplinary processes increasingly penetrate into society until the biological dimension of the reproduction of the population is reached, giving rise to biopower.

Biopower is the concept that Foucault uses to describe the impact of interprofessional relationships on patient autonomy. This autonomy is conditioned to the extent that professionals use the patient to communicate with each other, control the patient, and become a passive subject. The professionals determine the movements, spaces, and times dedicated to the patient. They exert their power on the patient through different manipulation mechanisms, or through what Foucault called “microphysics of power” [8,9].

The fundamental problems of biopower are how to monitor the patient, how to control their behaviour, their abilities, how to increase their performance, how to multiply their capacities and how to place them in the most useful place. The population is not only understood as a large human group, but as living beings governed by biological processes and laws that can be framed in health and development rates. Therefore, biopower reduces individuals to measures and figures manipulated by the health bureaucracy. The distinctive characters of the individualities and the advantage of the abstract that can be easily derived from any ethical evaluation are lost. To improve these situations, many health centers have launched strategies to humanize care to promote patient participation.

Biopower aims to clarify, measure, appreciate, and rank interprofessional relationships on patient autonomy according to the norm, in addition to being understood as a social and historically constructed production. This function of social demarcation is the main characteristic of the norm, governing at the anatomopolitical and biopolitical levels. Its main effect is the articulation of a normalizing society, of a normalized patient [10]. Biopower limits the ability to self-form subjectivity to the model of self-knowledge and identity. Therefore, the professionals who participate in this biopower configure subjects influenced by the domination of certain practices that determine the decision-making capacity of patients and themselves. To resist this appropriation of human life by biopolitical forces is to reinvent the space of ethics.

If we compare Foucault’s premises with the current situation of patients, the role of the patient in the clinical setting is usually seen as that of a passive subject [11]. Arrollo Arelano [12] built the concept of professional dominance exerting a totalitarian role on the patient. Thus, the traditionally “good patient” is one who assumes a strictly passive role, not questioning or protesting, and obeys all commands.

On some occasions, the exercise of power is clearly visible through the use of force or coercion. Some authors distinguish between the power exercised by doctors and that exercised by nurses. For example, doctors wield power in a clear way relative to the rest of the team in all situations that arise in daily clinical practice [13,14]. Hansson et al. [15] referred to the power exercised by the doctor as the medical status quo within hospitals that affects interprofessional relationships and patient care. These authors showed how doctors mark the times of clinical activity and influence the nurse’s ability to actively participate in the decision-making process in patient care.

At other times, this power is less obvious, since it is exercised subtly when influencing others through persuasion and manipulation or when occurring at a personal level [16]. The latter is considered by Hallet et al. [17] as a form of paternalism, where the nurse sees the patient as a subject that must be limited to ensure the therapeutic indications are met. Kottow [18] and Kleiman et al. [19] called the paternalistic model an active/passive model, and argued that the passive role of the patient is due to the belief of professionals that many patients, or most of them, do not have the educational and cultural background necessary to engage in an egalitarian relationship, thereby exercising more subtle power.

These authors also distinguished between an authoritarian paternalism, which imposes a hierarchical will on subjects who have autonomy and would prefer to make their own decisions, and a protective paternalism, legitimately exercised by the nurse toward a patient unable to make decisions because of their state of health. Forms of knowledge and institutionalized practices are established, which people naturally accept [20].

Several authors argue that the patient should place all their trust in professional criteria [21]. The patient trusts the nurse and lets them decide about the patient’s own care, which sometimes causes the patient to be treated by the nurse as a child. Many authors see the origin of this role in the Hippocratic Oath, which place excessive emphasis on power, knowledge, and professional virtues, without any mention of patient autonomy [22].

Henderson [23] stated that the professional who feels that the patient cannot make decisions about their own healthcare plays the role of the dominated subject. This situation causes a depersonalization of healthcare, and consequently a worsening of the relationship between nurse and patient [11]. Foucault [24] describes three instruments of domination applicable to the clinical environment (Table 1).

Different types of power in interprofessional relationships exercise strategies of domination over the patient, as identified by Foucault [25]: disciplinary, pastoral, self-government, and resistance power.

Disciplinary power creates a useful and docile subject that can be subjugated, transformed, and re-socialized [26]. This power maintains a status quo and new mechanisms of subjection and normalization are formed [27]. Studies showed that in clinical settings, interprofessional relationships are a dynamic of disciplinary power of obedience and dependence from the nurse to the doctor. Nursing has been considered in the epistemological ranks of professional knowledge as a minor science [28], leading to marginalization and the maternal stereotype of the nurse, compared to the dominant science of medicine [29]. These aspects help explain why the doctor can be defined by the nurse as the professional expert, who makes decisions about the criterion of the patient [30]. Even the PubMed/Medline thesaurus currently defines a health care team as a multidisciplinary team functioning in the care of patients that is typically organized under the leadership of a doctor (PubMed accessed January 2017). This definition maintains the dominance of the doctor as a leader, like a disciplinary power.

Pastoral power is another form of less taxing power that is based on information and knowledge, obtained based on a relationship of trust, emotions, or therapy. This power is an individualized form of power, in which someone acts as a guide for others [31]. The leader exercises power through duty and self-denial, because everything they do is for the sake of the “herd” [32,33]. Dingwall and Mclntosh [29] with other authors [34,35] considered the duality of the paternal stereotype of the doctor, whose goal is curing, and the maternal stereotype of the nurse, with a more affective value on the care provided, in response to the definition of pastoral power.

The strategies of self-government, also called self-management or subjectivation, are internal forms of power relating to oneself that become part of everyday life, not externalized, but in which knowledge and control relationships are involved [31]. Self-government is a form of power self-applied by individuals, directed toward the control of their own behaviour. Several studies [28,29,30,35,36,37] stated that the roles of other professional groups are influenced by the system of personal and professional beliefs of the group to which they belong. Elston [37] defined physicians as a social and cultural authority of judgments and definitions accepted as the truth. King [38] noted how physicians are automatically seen as the leaders of the healthcare team. Additionally, to Schön et al. [39], professional stereotypes reflect the epistemological ranks of professional knowledge, qualifying professions as greater (e.g., medicine) or lesser (e.g., nursing).

Finally, where power exists, so does resistance [40,41,42,43]. Strategies of resistance or struggle are present everywhere within the power net [44]. Resistance should not be conceptualized in terms of negation, but as a process of creation and transformation. At the moment in which a relationship of power exists, resistance is possible. We are not trapped by power; modifying your domain under certain conditions and according to a precise strategy is always possible. The purpose of this power is to infiltrate ever more deeply into human existence, both individually and collectively [44].

Some studies [13,29] have identified the boundaries, tensions, or resistance in the status quo of the group and the impact on patient care. Factors that limit resistance include the lack of training of professionals to face new challenges, changes in professional roles regarding group participation and communication, and rigid positions within the group. Depending on the professional’s degree of participation in decision making, professionals sometimes adopt more ambiguous positions and prefer that other professionals make the decisions normally made by the physicians. However, Björkdahl [45] and Ehnfors et al. [46] and others [47,48] observed that although some strategies are available for achieving teamwork, situations still exist in which professionals provide a false impression of the existence of a team through the use of rhetorical devices such as “we” in their description of daily professional practices, even though the reality is quite different.

Foucault [44] emphasized the inevitable role of the resistance in creating a discourse that necessarily results in counter-discourses [4]. This resistance is seen in patients labelled as “bad” or “demanding” patients. They are patients who resist and do not obey the orders of professionals, refuse the imposed treatment and care, and demand large amounts of information. This is, from the Foucaultian conception, a patient of the Resistance.

Foucault proposed using ethical archaeology to extract, record, and describe the statements that determine certain norms and rules. This archaeology helps analyse the discourses that define the autonomy of the patient in decision making. The discourses refer to the set of knowledge that legitimizes certain ways of acting and being [44]. The discourses refer to what can be said, thought, and done, but also to whom, when, and with what authority a person can speak [49]. For Foucault, the discourses have an institutional basis, that occur in the healthcare institutions [43,50]. These institutions play a key role in social normalization, being responsible for promoting this set of beliefs, narratives, and senses [50,51].

Through these mechanisms, dominant discourses are configured and ingrained, meaning they are considered unquestionable and provide a framework for discussing the value of speaking in one way about reality above others. These discourses can be considered regimes of truth, since they determine what counts as important, legitimate, and relevant knowledge [4,41,52]. These are forms of normalized and legitimized systems instituted to shape the practices of professionals [52]. As such, discourse can be seen as a unit of map analyzing professional conversation sets as a repetition of behaviours over time and space [53].

Pinho and Azevedo [54] claimed that nurses, when speaking, understand that the relationships established among the teams are important to the satisfactory performance of their duties. Nurses maintain an interest in cooperating with the team, with the goal of being co-responsible for patient care. However, nurses have admitted that this is not always true. Riley et al. [55] and Benner [56] revealed that the discourses of nurses may appear as merely subordinate to the doctor’s decisions. Little et al. [36] indicated this position as limiting the fulfilling of the patient’s needs and their decision-making ability.

Zaforteza et al. [57] concluded that doctors have a speech based in intransigencies in maintaining their hegemony in decision making, information provided to the patient and family, and the establishment of standards in services. These factors cause harm to the patient and family in their care because they will not benefit from all the knowledge and skills of the nurse.

Finally, although this article focused on the analysis of Foucault’s perspective on the subject’s autonomy, we note that many authors consider it is possible to take advantage of the expert knowledge of the professional to help patients reflect and adapt their preferences as part of autonomous decision making, without falling into fixed, standardized, and institutionalized norms [58]. The creation of climates and the skills and individual virtues of health professionals can develop the capabilities of patients [59]. McKenzie and Stoljar [60] emphasized the independence of patients and explained how the social-historical context influences the ability of a person to make decisions in all dimensions of their lives. They concluded that cultural norms, structures, and social practices affect the identity of an individual, and therefore their capacity for autonomy.

Some international guidelines [61] have attempted to increase the autonomy of a patient by proposing communication strategies between professionals and patients, and improvement in individualized care. Strategies that promote standards providing a legal framework and involving the patient’s family in decision-making, also promote respect for dignity in accordance with the wishes and intentions of patients.

The main objective of this study was to analyse the impact of the interprofessional relationships on the decision-making capabilities of patients from the nurse’s perspective using Foucauldian ethics.

This objective is important for several reasons: (1) the reviewed literature did not provide an in-depth analysis of the power relations that legitimize certain professional practices and dominate the decision making of patients in the clinical setting; (2) a limited number of studies have analysed interprofessional relationships from nurses’ perspectives; (3) studies describing the role of patient autonomy in decision making about their own health care are rare; (4) few studies have critiqued the current application of ethics from Foucault’s perspective.

## 2. Materials and Methods

### 2.1. Research Design

A qualitative study, based on poststructuralist references of the ethical theory of Michel Foucault, was conducted. A discourse analysis was conducted through 13 in-depth interviews with nurses from internal medicine and specialty departments in a general hospital.

### 2.2. Participants and Research Context

In the hospital analysed, the Department of Internal Medicine and Medical Specialties has a total of 60 beds with an annual patient occupancy of 92%. These services, compared to the other units, have the highest health care burden per year and serve the most range of diverse pathologies.

With respect to professionals from both services, we highlight the following characteristics: the nurses, including 23 regular staff and approximately 70 temporary employees per year, with an average age of 33 years, a minimum experience of five years in the department, and nine years as a nurse, who work three shifts in the morning [4], afternoon [2], and night [2]. For these services, most nurses work in medical specialties.

The reasons for selecting these units were as follows: (1) these are the departments with the highest occupancies at the hospital. This factor may result in different types of management of the health care load, staffing, and relationships when compared to other hospital departments that have provided more information on this phenomenon; (2) these departments care for patients with diverse pathologies, providing insight into the various types of care provided to patients, depending on their complexity; (3) these departments have the highest health care burdens. Therefore, the possible tensions and resistance as a result of the professionals’ workloads were analysed; (4) these departments are associated with a greater number of professionals on staff but a lower nurse–patient ratio and a greater diversity of the specialties involved in patient care.

### 2.3. Interviews with Nurses

Of a total of 16 nurses in the two selected departments, only 13 met the inclusion criteria. Ten semi-structured interviews were conducted to ensure the level of theoretical saturation was reached. The inclusion criteria were: (1) nurses with a minimum experience of five years, which is considered enough time to have some professional expertise in the development of nursing care; (2) nurses with a minimum tenure of three years in the departments under study, which is enough time to understand the structure and function of this unit and the professionals involved in the team; (3) nurses who agreed to participate and signed the informed consent.

### 2.4. Data Collection

An in-depth interview was completed through non-intrusive or direct, yet comprehensive interaction of the participants within the framework, observing reality as they experience it and how they view the phenomenon being studied. The entire data collection process was accompanied by the development of a field diary and dual digital recording in the presence of an observer who collected data on metalanguage, positions, gestures, etc. The shortest interview was 34 min and the longest was 68 min. Each recording was incorporated into the database for the study, for the sole and exclusive use of the research team members. After checking the audio, the recordings were delivered to the transcriber, who had previously signed a confidentiality agreement regarding the data. To ensure data anonymity, each transcript was reviewed, and symbols were substituted for the names of people or places. From then on, every interview was assigned a hermeneutic unit by the qualitative program ATLAS.ti version 6 (ATLAS.ti Scientific Software Development GmbH, Berlin, Germany).

### 2.5. Data Analysis

Discourse analysis was used to explore the patients’ decision-making capabilities and how this has been configured in the health context, based on interprofessional relationships from the nurses’ perspectives. Discursive analysis was used to reconstruct the meaning of the text from the specific to the general. Association and interpretation, along with the extraction of conclusions, involved interpreting the transcripts and their implied significance based on Foucault’s poststructuralist theory.

Foucault’s ethics explains the dimension of the relationship with actual conduct and codes or systems of prohibitions, requirements, and assessments. This determines how the individual conducts themselves as a moral individual, which shapes the modes of subjectivity [38]. So, we wanted to find the connection between patient autonomy and the exercise of professional power by analysing the relationship established between these professionals with the surrounding context [39]. Therefore, we analysed the mechanisms and procedures present in the exercise of power, through standardization, homogenization, impositions, and oppression strategies that operate in relation to professionals and the professional practice environment. This will provide a vision for the guidelines, rules, and regulations that govern professional practices from the nurse’s perspective.

Foucault ethics attempts to analyse professional relationships based on codes that currently dictate what behaviours are permitted or prohibited in practice against non-authoritarian and unified personal choice, opening a new avenue for understanding ethics [40]. The criteria used to assess the methodological rigor of the study were credibility, auditability, and transferability in accordance with Morse [41] and Guba and Lincoln [42].

### 2.6. Research Limitations

Given the scarcity of international studies that relate the perspective of Foucauldian ethics with patients’ autonomy in decision making, comparing this study with other studies in similar contexts was not possible. This research has a voluntary restriction because sex was not considered, as this study focused on power relations and not specifically on the profile of professionals in the health care model that, until recently, has been doctor-man and nurse-woman. Another limitation of this study is that not everything was analyzed in the discussion of doctors and patients, so certain aspects could have contributed more depth.

### 2.7. Ethical Considerations

This research involves confidential data and sources, from the personal data of patients to the professionals who treat them. This study was approved by the Ethics Committee for Clinical Research of the Balearic Islands, with the code IB1561/11, and was authorized by the hospital. Participants received information about the research and signed an informed consent document developed for this study. We respected the legislation in Spain and the principles of the Declaration of Helsinki and other international recommendations with regards to data protection. No conflicts of interest existed between participants and researchers conducting the study, and we considered the ethical implications of this study at all times.

## 3. Results

The interviewed nurses were two men and 11 women, ranging from 29 to 54 years old, with between 9 and 28 years of professional experience. Some of the participants had been in the department for more than three years; they had been permanent staff for only a few months. Notably, the interviews were conducted during a period when the country’s economic crisis imposed major austerity policies on the health sector for both resources and personnel, which appeared in the nurses’ discourse.

Many factors involved in interprofessional relationships were detected, as well as differences in discourse based on professional experience, age, years of service and unit. Relationships based on hierarchical models according to professional categories, where each of the involved agents built their own professional stereotypes and those of the other team members, dominated the discussion. The nurse depends on the doctor, who is considered an expert and the highest link in the chain of command. In turn, the nurse’s assistant depends on the nurse. Based on these categorizations, the roles of each of the team members were articulated, demonstrating the influence on clinical practice and the relationship established with the patient and family. Interestingly, the patient constantly appears in the definition of teamwork but only as a passive element that is used by every professional to communicate with the others, and even to confront each other.

From the results obtained in this study and after discourse analysis, interprofessional relationships were defined, especially between nurse and physician, from the perspective of the nurse. So, from this analysis of the interviews, we created a category called the power of the health care team to make decisions for the patients, with seven codes defined from the discourse of the nurses: idealization of teamwork: practice realities; nurses are like the ‘glue’ of the unit; communication and a lack of support; professional stereotypes: expert doctor, obedient nurse, and submissive assistant; and impact of interprofessional relationships and teamwork on patient autonomy. Table 2 defines the codes along with the most significant textual phrases of the participants.

## 4. Discussion

Foucault attributes the subject to the question [1], criticizing the notion of the subject as a category and as an oppressed empirical entity. This is shown in our research through the domination exercised by the professionals. We demonstrated that the analysis of the decision-making capacity of patients is conditioned by the professional’s interaction with the patient and family, and also by the relationships between the professionals involved in healthcare, as shown by the study findings and most studies on this subject [45,46,62,63,64]. The description of the strategy framework in the interprofessional relations confirms the presence of the biopower device described by Foucault. The risks that affect this current rationality, that insists on the appropriation of life under biological codes [1], require attention.

The presence of these power strategies over patients is contrasted by Kantian ethics, to a postmodern Stoic ethics, denying the foundation of a moral subject, which criticizes the universal categories and the supposed essence or foundation of the moral subject. Foucault validates the idea that the subject is an absolute product of the relations of power, knowledge, and morality [64], supported by the results of this study. In Foucault’s words, “fallacious the necessary universality of any ethical proposal” [6] is evidenced in the research, but does not work according to current ethical models.

The results revealed that power is all around us, affecting patients, professionals, and health institutions, and power plays have an important role in all relationships. This was observed in the lack of teamwork due to the interprofessional relationships in a hierarchical structure with vertical power and ineffective communication flows. Considerable evidence supports the idea that interdisciplinarity in the health care system is a fallacy that pervades the institutional discourse but is not practiced in reality. This study shows little professional adherence to this practice occurs [57,65]. This explains the paradox of teamwork reported in studies by Björkdahl [45] and Ehnfors et al. [46], where a cultural conception of “we work better together” is not efficient in the clinical setting, as explained in the analysis of the discourses in this study.

Foucault argues that the various and multiple forms of repression are globalized from the perspective of power. A dominant discourse explains that the nurse depends on the doctor’s decisions in almost all parts of the nurse’s professional practice, and the patient would be at the bottom rung of the decision-making ladder. Nursing has been considered in the epistemological ranks of professional knowledge to be a minor science [63], leading to its marginalization and the maternal stereotype of the nurse, in comparison to the dominant major science of medicine [35]. The strategies here were to determine the use of time, the regulation of cycles, or the establishment of individual and collective rhythms. The power flows are perceived differently, unequally, or asymmetrically in interprofessional relationships, which are standardized and promoted by the health care institution [13,15,57,66,67].

Therefore, as shown in this research, professionals exercise disciplinary power through a series of mechanisms of biopower that affect the autonomy of the patient such as standardization, homogenization, monitoring and control, subjugation, the clinical gaze, control of spaces and the use of times, and rewards and sanctions [34] (Table 3).

Criticism of Foucault tries to question the relationship between power and knowledge, and how this relationship is embedded in daily practices [68,69,70]. This perspective helps to generate new positions from which to resist, challenge, or transform dominant discourses that are accepted without criticism or reflection, and that perpetuate professional stereotypes [71]. An emerging discourse appears in this study via a nurse who proposes resistance to the doctor’s dominance to reduce the asymmetry of power between the two. Such resistance is materialized by a subtle exercise of power by advising the doctor about certain clinical decisions. However, forms of resistance in patients are displayed in those who do not obey orders and demand greater prominence in their care.

The most pervasive effect of these power relationships on the patient is the result of a subjectivity annulled by the possibility of self-construction, which underpins the patient’s identity within the parameters of the scientific understanding of self [52]. Therefore, the patient in the clinical setting, between the force or power of being affected and the function of some professionals’ strength or power to affect others [3,31], intersects with the biopower [54] that results in the anatomical and biopolitical appropriation of the body. In the context of health care, where it has been shown that power is an integral part of the daily activity of clinical routines, biopower legitimizes a system that identifies the production of individuality through a web of power relations [55].

**Figure 1:**
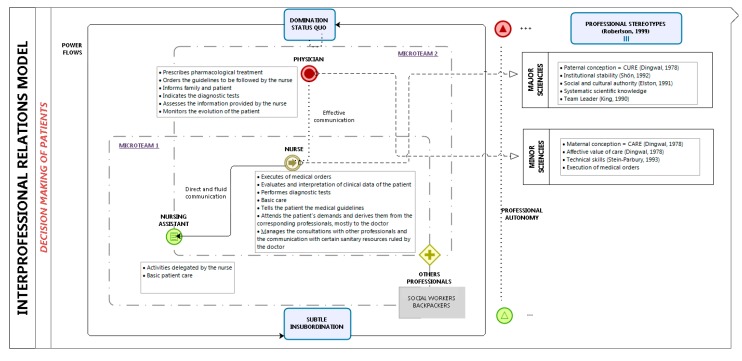
Interprofessional relations model in decision making of patients.

Figure 1 shows a model of the impact of interprofessional relationships in patient autonomy from an analysis of the outcomes of this study with other similar research.

However, the ethics proposed by Foucault presents some criticisms that are still unresolved as seen in Table 4.

## 5. Conclusions

The analysis of the results shows that the patient must be placed in a historical and plural horizon in which universality and normativity are no longer required. The results establish a struggle where power relations have become a network that includes both professionals and patients. To understand morality beyond a legal code, which treats subjectivation a submission to the norm, morality of self-practice yields where the code is blurred and relationship is established with oneself.

The transformation of the individual’s mode of being is equivalent to the change of a variety of relationships: with oneself, with others, and with the truth. This transformation is a question of going beyond a model of individualization, the frontiers that draw the standardization devices, to open a space to new experiences through a relationship that the individual realizes with themselves and with their limits. Therefore, ethics is a practice of resistance that recreates the relationship of strength, finds vanishing points and tense areas that appear vulnerable, and travels the limits imposed on us.

Self-care is a lifelong practice that ensures the continued exercise of freedom. Self-care is about liberating ourselves from the imposed rules, the modes of subjection, to access our own behaviour or technique of subjectivation. That is, to self-care affects one’s own lifestyle.

So, generating effective communication channels that allow patients to fully access information about their disease process is essential for autonomous decision making. The nurses’ position should be more effectively used, as they argue for an egalitarian model of teamwork beneficial to the patient, thereby abandoning the idea of a team where the patient and family are constantly excluded from decisions about their care.

## Figures and Tables

**Table 1:** Strategies to control patients [24].

Strategy	Description
Surveillance	Strategy of control and production of behaviours that automatically occurs in the patient through an absolute coercive look exercised by professionals. The French philosopher calls this Panoptism.
The normalizing sanction	Infraction that sanctions anything that does not conform to the rule, to the indications of professionals, reducing the possibility of deviation or difference, hierarchizing the value of patients’ capacities, or tracing the limit of the abnormal. This technology forces homogeneity to reject everything that escapes the norm and labelling the patient as a “bad patient”.
The examination	Based on a system of objectivation that makes individuality enter a documentary field as if it were a describable and analysable unit, thus explaining the biomedical or biologicist model in health institutions. The hospital has required practices and operative speeches to make effective the production of disciplined individuals.

**Table 2:** Category, codes, and definitions of interprofessional relationships in the decision making of patients, for the category of the power of the health care team in making decisions for patients.

Code	Definition	Verbatim
Idealization of teamwork	The concept of teamwork stands out as diffuse, without clear characteristics. The nurses, rather than defining a joint effort with all team members, describe micro-teams.	E1: “*In my unit, I think that many do not work in a team. I mean, we complain about each other, but, in the end, nobody does anything to improve teamwork…*”
	The doctor is considered the top person on the team. Medical practice is highlighted as a priority, relegating the other nursing activities. Decision-making within the team is attributed to the doctor.	E6: “*That is ideal. The practice? In my service? We do not go with them. Here, everyone moves freely. I try to go with the oncologist, but I do not always succeed.*”
	A leading role for nurses for better communication between team members is proposed as key to the team’s smooth operation. It is a theoretical work model that has not been achieved in hospital units.	E6: “*What they told us in school. This would be ideal...previously they had a session where it was discussed; they spoke of the cases, the cases that are logged...the view of the nurse would be requested...and then, you, the doctor, nurse, would go to see the patient.*”
The nurse in the healthcare team	The doctor is identified as being responsible for providing information. The nurse expands the information due to the short amount of time the doctor spends with the patient. For nurses with more years of experience in the unit, greater concern about the patients’ wellbeing is observed in their discourse due to problems with team members.	E5: “*…sometimes we even have to respond to things from the doctors, also, things to cover their backs, but there are things that the doctor has not informed us about or when they happen, or what it is called, or...the patient is well informed but...there are fewer questions.*”
	The nurse assumes a strong role of supervision and control of the proper functioning of the unit and patient care. All nurses share the idea of being a key element in the functioning of the unit.	E1: “*The nurse does have an important role. Yes, because I think if it weren’t for the nurse, things would not work as well, I mean, because you’re attentive, everything else works because even if everyone does their part separately, it is the nurse who later does it all, who monitors the work of everyone else.*”
	The doctor’s dependence dominates. Decision-making about the patient, even about basic care, follows the doctor’s indications. Major communication gaps occur between both professionals, causing conflicts with the patient. Nurses with more years of experience, although they assume their role as dependent on the doctor, exert power in a subtle manner.	E2: “*The doctor is the one who makes medical decisions and, without them, we do nothing, right? But, because there is so little communication (laughs), in the end, what happens is that we are a bit lost.*”
Limitations on teamwork	There are not adequate communication channels between the doctor and the nurse, causing an increase in workload due to efforts to re-channel information between them. Notably, for nurses with more experience in the unit, poor communication with the doctor does not cause a reactive and frustrated attitude as it does with less experienced nurses, but they instead opt to resign and establish lines of communication with the doctor outside of protocol.	E1: “*…often, after the doctor visits for a little bit, you have to call because you have a question...Then, of course, if the doctor talks to you and says look, I am going to ask for a radiograph or something, or I’ve changed the antibiotic to this, you would not have to call later…*”
	The nurse would much rather support the assistant, and the assistant would choose a more involved nurse in the delegated activities.	E10: “*I would say that the assistants in my service could help with nursing a little more…*”
	Professional experience, seniority, and experience level in the unit are sources of tension within the healthcare team. The rejection of newer professionals was noted, especially by nurses with more experience. Nurses with less experience in the unit define the older and more experienced nurses as being confined to historical practices and standards.	E2: “*…The more senior you are, the tension you causes about small things...I guess they are like work habits that you have...The less time you have worked, the fewer customs you have stuck in your head, and then it affects you less*.”
	Limited professional empathy for other professionals. The lack of consensus on care and the lack of nurse satisfaction in the service provided also appear to be factors that hinder teamwork.	E5: “*They are good workers, yeah, very good, but they lack such training, they lack empathy with patients, the family, with their partners, and if a person doesn’t empathize there, they don’t empathize on the street, or at home, or anywhere else…*”
	The workload results in few opportunities and spaces in which to work collectively. We perceive that a structural change in the organization would be required to create more available time.	E7: “*…you want to do all the work, which is a lot, and sometimes you can’t, you don’t manage to, and that creates tension because of course we want to reach the end of the shift and leave everything perfect, and you can’t most times, and then this generates more work than the previous shift…*”
Professional stereotypes: expert doctor, obedient nurse, and submissive assistant	The nurse describes the doctor as a professional expert, upon whom they are dependent, and is sometimes considered to be outside of the team. This stereotype of the expert doctor, based on the biomedical model established in the healthcare system, is characterized by a lack of communication, limitations placed on nurses in making decisions under the doctor’s judgment, and limitations on the time spent by the doctor with the patient.	E1: “*I always think the biggest expert is the doctor...Well, I think all of us because the doctor says one thing we’re going to do, we must do rinses or we should try to make postural changes, well, although the doctor says it, we all know what we need to do.*”
	The nurse has a submissive stereotype based on the hierarchical relationships of the doctor’s dominance in clinical practice, which means that the nurse assumes a series of delegated responsibilities and acts merely as executor of the doctor’s orders.	E2: “*Then, comes the doctor and sees him for five minutes…The doctor is the one who makes medical decisions and, without them, we do nothing, right? But, because there is so little communication (laughs), in the end, what happens is that we are a bit lost.*”
	The health care institution is referred to as an excessively hierarchical organization that distributes workloads and the ability to participate in the centre’s decision-making based on professional categories. This situation causes inflexible stereotypes that are resistant to change, and creates situations that limit the decision-making ability of patients.	E8: “*…Sure, doctors get along with everyone, nurses get along with everyone except the doctors, the assistants get along with, well, get along with nurses well but better with the guards, so to speak, right? It depends on the categories, right?*”
Operation of micro- teams in the healthcare team	The micro-team, formed by the nurse and the doctor, is based on a relationship of trust, with large deficits in communication. The main axis is the doctor. The organization of the nurse’s work is determined by the doctor’s agenda and the performance of standard diagnostic tests. The doctor only recognizes basic care as being the nurse’s partial responsibility, as this is also susceptible to medical decisions in the case of complications.	E1: “*During the morning shift, the doctor has to do consultations and he has very little time to see patients on the ground; then, they often have to trust what we say.”*
	Nurses consider the micro-team formed with the assistant to be fundamental. The relationship is based on trust and closeness. The goal is to share information on patient care, where the nurse’s perspective prevails.	E8: “*When I begin the shift with the assistant I like to go over if there is something important that is not there because it can be there on the tray and maybe I think it’s their job and they will do it, but, as a human, they could forget…*”
The patient as a communication tool between team members	For fluidity in communication, it must come from the nurse and depends on the doctor’s attitude. A sense of resignation appears in the nurse if communication with the physician is insufficient.The nurse and the assistant have a more direct relationship. Communication flows are established during shift changes, in the patient rooms, and the nurse’s station.Between nurses, direct communication occurs, and they share information or concerns regarding patients.	E7: “*There is very poor communication both by doctors to the patient and family as well as by the doctor to the nursing team, and, as a result, there are very noticeable failures because sometimes the patient knows things we do not know and we seem unprofessional…*” E5: “…*for example, I tell the assistant everything at the end of the shift. Sometimes she says, why are you telling me this? In case you want to know. And I tell her all changes and anything at the end of the…*”
Impact of interprofessional relationships and teamwork on patient autonomy	Communication difficulties in the team cause communication with the patient and family to be poor and create confusion due to the lack of consensus among the professionals.	E3: “*Well, it’s bad...Maybe with the way we work, we convey it to the patient, and the patient is not to blame for anything, but the patient perceives it, and it can create discomfort for the patient…*”
	The lack of teamwork causes patient discomfort and repetitive activities, as well as failures and errors. In cases of non-fatal errors, they respond with corporatism to avoid patient mistrust toward professionals.	E2: “*It is not the same as going every man for himself; for example, the assistant does hygiene, leaves you the patient to cure the ulcer, you have to go by yourself...rather than everyone go together, you use three steps, and I think that affects the patient, clearly.”*
	For the nurse to feel safe and supported, they need a competent work partner and to work in teams, or at least to collaborate. Without this relationship, the degree of professional satisfaction decreases and impacts the quality of patient care.	E2: “*It is not the same as having a teammate that you know will support you, who will help and you feel safe because you don’t have a partner you have tension with, I suppose because then you feel insecure, you feel, I don’t know.*”

**Table 3:** The impact of the mechanisms of disciplinary power on patient autonomy.

Mechanisms of Disciplinary Power	Description	Impact on Patient Autonomy
Normalization strategies	Common definitions of objectives and procedures that manifest in how you should arrange and organize professional activity. Its purpose is for professionals to be included in and identified with certain standards, achieving conformity within a health structure.	Standardization strategies define what is normal or deviant, accepted or unacceptable, superior or inferior, good or bad, directly or indirectly affecting the decision-making capacity of patients.
Homogenization	The mechanism of power verified in this research that hinders the individuality and uniqueness of our patients.	Modelling a type of patient passivity, or what Foucault called passive subjectivity in relation to oneself, because the patient is guided and directed to take charge of a truth provided by professionals that is virtually assumed to be accepted. Truth is thus configured as an element of the genealogy of ethics. The truth is related to power and it carries mechanisms of submission. In addition, it has effects on the individuality of patients [50].
Surveillance and control	Foucault [26] pointed out how, through vigilance, whether deliberate or not, practitioners exercise their systems of control over power and knowledge.	Determine the most strategic positions of those thought to be inferior, such as the position of the doctor on the nurse and the position on the patient.
Subjugation	Physical and symbolic strategies that involve the individual in such a way that their movements and rhythms respond and are subordinated to the needs of the disciplinary devices. The subjection of patients to certain guidelines, rules, or norms is fundamental for sustaining the power relations that govern the health institution [43].	The strategies of subjugation to the patients observed are mechanisms of imposition, subjection, repression, oppression, and dogma.
The clinical view	Metaphor that Foucault used to refer to another power strategy where events are read, organized, and interpreted in an anatomical-clinical conception [24].	Extrapolated to an everyday view that is inscribed in clinical context and is both an effect and supports certain practices and relationships with patients.
Control of spaces and the use of the times	The control of spaces is the distribution and allocation of patients and interprofessional relationships to certain spaces, often spaces of closure. For Foucault, both physical and symbolic spaces are a fundamental piece for the device of knowledge and power.	The use of time is a strategy of exercising power by fragmenting or dividing activities or tasks at fixed times and pre-established times, which becomes a new control device.
Rewards and sanctions	Are strategies through which the permanence of an order or a normative power is achieved.	The management of rewards and punishments or threats according to the consideration of good or bad patient are achieved some of the mechanisms discussed above and reflected in the results.

**Table 4:** Criticisms of the Foucaultian ethical proposal.

Authors	Criticism
Molina-Mula et al. [72]	The assertion that Foucault’s ethics is a return to the subject matter that is solved with a new ethical approach, as opposed to the theory of the constituent subject involving a new conception of subjectivity.
Taylor et al. [73]	Believes that Foucault silences the moral foundations of his theoretical options and does so because they are humanistic criteria that he himself has rejected.
Rochlitz et al. [74]	Points out that Foucault’s critical interventions are norm-bearers and virtually universalist, since they refer to a demand for autonomy of the person and opposition to unjust suffering.
Hadot et al. [75]	Focuses his criticism on the incorrect use of historical material, considering Foucault’s ethical proposal as a personal bet rather than a faithful reflection of ancient ethical experience. It also considers that the practice of self-care without universal criteria necessarily results in an elitist scepticism that only applies to a few.
Habermas et al. [76]	Discusses Foucault’s ethics based on considering the existence of a self-referentiality, and an absence of normative foundations that designs a political theory without justification, where the lack of response to the ultimate meaning of resistance condemns the proposal to an arbitrary decisionism.
